# Pleuro-Pneumonia, with Subsequent Scorbutus

**Published:** 1877-09

**Authors:** D. W. Scott

**Affiliations:** Castlemount, Ga.


### PLEURO-PNEUMONIA, WITH SUBSEQUENT SCORBUTUS.

By D. W. SCOTT, Castlemount, Ga.

I was called, January 19, to Nathan ——, æt seventeen, and found him with considerable dyspnœa, so much so that elevation of the shoulders was frequently necessary, and with excruciating pain in the right side of the chest. I learned from his friends that two days previously he was suddenly attacked with chill and marked rigors, followed by high febrile excitement.

After careful examination of both lungs, it was evident that the right lung and pleura were involved in active inflammation. Some soreness of the mouth also existed, which I could not at the time account for. His appearance indicated extreme anxiety and suffering; pulse was moderately strong and numbered about one hundred and twenty beats to the minute.

The usual treatment for similar cases was adopted, such as veratrum, to control the heart's action, and thereby lessen the amount of blood carried to the engorged and inflamed parts, blister on the affected side, calomel, etc. The pain was lessened at once by opiates, and the unpleasant symptoms measurably controlled permanently, after the sedative and blister had acted.

Under this course of management the patient was soon convalescent, and on the 24th, five days from my first visit, and about one week from the attack, he was dismissed, as I supposed, free from danger of further difficulty. Ten or twelve days subsequently, I was again called to arrest persistent epistaxis. Applications by injection, and the tampon, of tannin, muriated tincture of iron, etc., gave no permanent relief from the hemorrhage, and indeed other parts, such as the gums and mouth generally seemed to take on a hemorrhagic disposition, with apparently fungus granulations on the tongue.

I learned from the patient that shortly after being relieved from the pneumonic attack, he was troubled with intolerable itching over the whole surface of the body, which subsided as the hemorrhage from the nose and other parts commenced. The hemorrhagic condition continued till the patient's death, on the 15th of February, one week after being called the second time, and nearly a month from his attack of pleuro-pneumonia.

As to the origin and nature of this scorbutic or hemorrhagic condition, I am at some loss to determine. I have not decided to call it purpura hemorrhagica, but as scurvy will include other forms of depraved blood, think proper so to denominate it. Now, whether this state of the system led in any way to the acute pneumonic attack, or whether the scurvy was the result of debility caused by pneumonia, is more difficult to decide. Two facts must be taken into the consideration of this question: The patient, I learn, had been an inveterate salt eater. His food was made so salty that no one but himself could relish it; and the circumstance mentioned before, of his having sore mouth when first seen with pleuro-pneumonia. These facts seem to justify the opinion that, at least, the scorbutic condition was independent of the pectoral trouble, whether the former had any agency in the production of the latter or not.

